# Evaluation of follicular flushing with double lumen needle in patients undergoing assisted reproductive technology treatments

**DOI:** 10.5935/1518-0557.20210009

**Published:** 2021

**Authors:** Marcelo Marinho de Souza, Ana Cristina Allemand Mancebo, Maria do Carmo Borges de Souza, Roberto de Azevedo Antunes, Ana Luiza Barbeitas, Verônica de Almeida Raupp, Layna Almeida Barbosa da Silva, Flávia Siqueira, Ana Luisa Bruno Marinho de Souza

**Affiliations:** 1 Fertipraxis, Human Reproduction Center, Rio de Janeiro, Brazil

**Keywords:** ovarian follicle, oocyte retrieval, instrumentation, *in vitro* fertilization

## Abstract

**Objective:**

The purpose of this study was to investigate the possible impact of follicular flushing on the number of oocytes retrieved and oocytes in metaphase II in patients with poor ovarian response (POR) compared to direct aspiration.

**Methods:**

This prospective, comparative, randomized single center study included 208 punctures of patients with POR, submitted to assisted reproduction technology (ART) treatments. Two groups were compared; one in which double lumen needles were used (Wallace DNS1733) for follicular flushing (n=105), and one in which single lumen needles were used (Wallace ONS1733) for direct aspiration (n=103), upon the observation of ≤ 5 follicles between 15-17 mm, ≤ 4 follicles with sizes greater than 18 mm on hCG day, and ≤ 7 recovered oocytes.

**Results:**

There were no differences in age (39.07±3.88 vs. 38.11±3.43); weight (61.73±17.53 *vs*. 65.96±15.44); AMH (0.63±0.59 *vs*. 0.94±0.97); stimulation days (9.57±1.87 *vs*. 10.29±2.82); estradiol levels (788.94±670.82 *vs*. 940.16±694.69); progesterone (617.29±319.76 *vs*. 561.18±486.78); or number of follicles with sizes ≥18 mm (1.84±0.95 *vs*. 2.07±1.09). Although gonadotropin totals (1678.28±798.52 vs. 2080.45±852.36; *p*=0.0008), number of aspirated oocytes (3.00±2.11 *vs*. 3.69±2.20; *p*=0.02), and number of metaphase II oocytes (2.20±1.64 *vs*. 2.99±1.88; *p*=0.02) were significantly different, oocyte / follicle ratio ≥15 mm (0.93 vs. 0.98) and metaphase II oocytes / follicles ≥15 mm (0.68 *vs*. 0.79) were similar in both groups. The failure to capture was 16% *vs*. 9.8%.

**Conclusions:**

Considering that there was no difference in the oocyte per follicle ratio, follicular flushing did not increase the number of oocytes recovered from poor responders.

## INTRODUCTION

In assisted reproduction, oocyte retrieval is one of the key elements in highly complex procedures. Until the beginning of the 1980s, oocyte retrieval was performed laparoscopically, despite its inconveniences, greater risks and lower efficiency, until the emergence, in 1981, of transvaginal ultrasound-guided aspiration (Xiao *et al*., 2018; Roque *et al*., 2012). TVUS-guided aspiration soon became the standard method, due to its greater safety, effectiveness and ease of execution in assisted reproduction technology (ART) treatments (Kumaran *et al*., 2015; Lenz *et al*., 1981). In summary, the procedure is performed with the patient under analgesia, using an aspiration needle properly adapted to the vaginal transducer, through which the interior of the ovaries is accessed for the emptying of the follicles and retrieval of the oocytes. Two types of needles are available, of one or two lumens. In the latter, one of the channels lends itself to aspirating the follicular fluid and the other to inject its own follicle refill solution followed by a new aspiration, in what is conventionally called follicular flushing. The purpose of this procedure is to increase the potential for success with possible maximization of the number of oocytes recovered (Roque *et al*., 2012).

According to Pellicer *et al*. (1987), poor ovarian response (POR) is observed in approximately 9% of the patients submitted to ovarian stimulation treatment, although wider variations, from 10 to 30%, have been reported in medical literature (Pellicer *et al*., 1987). It is well understood that POR is an entity ascribed different definitions, with emphasis on the Bologna criteria (Ferraretti *et al*., 2011) and, more recently, a new more detailed stratification of low responders to ovarian stimulation, the Poseidon Group (Pacient-Oriented Strategies Encompassing Individualized Oocyte number)(Poseidon Group, 2016). Poor response to gonadotropin stimulation results in the retrieval of a fewer oocytes and embryos for transfer, ensuing lower pregnancy rates in in vitro fertilization (IVF) treatments (Ulug *et al*., 2003).

Observational and nonrandomized studies have suggested that flushing may result in a greater number of oocytes recovered (el Hussein *et al*., 1992; Bagtharia & Haloob, 2005; Mendez Lozano *et al*., 2008). However, this trend has been questioned in the last decade by randomized controlled trials (RCTs), which failed to demonstrate that follicular flushing improves outcomes in terms of number of oocytes recovered and mature oocytes and pregnancy rate (PR), when compared to simple aspiration without flushing (Levens *et al*., 2009; Kara *et al*., 2012). Currently, it seems clear that follicular flushing has limited benefit in normal-response patients, and should not be interpreted as a daily routine procedure, since it increases the duration of the procedure (Wongtra-Ngan *et* *al*., 2010). Additionally, with respect to patients with POR, recent data seem to point to the lack of consistency in recommending the procedure routinely, although it is still performed in some centers (Roque *et al*., 2012; Levy *et al*., 2012).

Thus, the objective of this study was to evaluate the possible effectiveness of follicular flushing for the retrieval of oocytes in patients with poor response to ovarian stimulation undergoing IVF treatment.

## MATERIAL AND METHODS

A prospective, comparative, randomized, single-center study was carried out from May 2017 to July 2018 at the Fertipraxis Human Reproduction Center, a private clinic in the city of Rio de Janeiro. The study included 208 punctures of patients with POR, submitted to assisted reproductive technology (ART) treatments. Two groups were compared: one using a double lumen needle for follicular flushing; and one using a single lumen needle for direct aspiration, upon the observation of ≤ 5 follicles between 15 - 17 mm, ≤ 4 follicles above 18 mm on the day of the hCG. The patients’ ages ranged from 34 to 42 years.

### Ovarian stimulation

Follicular growth stimulation was initiated between Days 2 and 5 of the cycle, with urinary (Menopur, Ferring, Germany) or recombinant gonadotropins (Pergoveris, Merck Serono, Switzerland), with individualized doses that varied from 150 to 300 IU daily, adjusted when necessary, according to the assessment of the attending physician and based on ultrasound monitoring of follicular growth. Once the minimum follicular diameter criteria described above were reached, a single dose of 250 µg of r-hCG (Ovidrel, Merck-Serono, Switzerland) was administered to induce ovulation and oocyte maturation.

### Oocyte retrieval

The procedure was performed 36 hours after hCG injection, with the patient sedated, with an aspiration needle attached to its own guide, properly fitted to the vaginal transducer. Aspiration was performed by emptying the follicles, in a closed-circuit system using an aspiration pump (Pioneer Pro-Pump OS 483) with pressure set at 90 mmHg. The follicular fluid was directly deposited in a 14 ml conical tube. In the follicular flushing group, 17-gauge double-lumen needles were used (Wallace DNS1733); after the first aspiration of each follicle, half buffered medium (PBS, Ingamed^®^) was injected into it, followed by a new aspiration, and the liquid was evaluated by the embryologist to identify the cumulus-oocyte complex. Each follicle was aspirated up to 3 times. For patients in the other group, single-gauge 19-gauge needles (Wallace ONS1733) were used.

### Statistical analysis

The data were expressed as mean ± standard deviation (SD); the mean number of oocytes retrieved from the groups with direct aspiration and with flushing were compared based on Student’s t test. *p*-values <0.05 were deemed statistically significant. Statistical analysis was performed on SPSS Statistics, version 22.0 (IBM Corporation, USA).

## RESULTS

A total of 208 ovarian puncture procedures for IVF / ICSI or oocyte freezing were evaluated and divided into two comparison groups, one using double lumen needles for follicular flushing (n=105) and another using single lumen needles for direct aspiration (n=103). Patients mean age was 39.07±3.88 years in the double lumen needle group and 38.11±3.43 years in the single lumen needle group. Other clinical characteristics are summarized in Table 1. There were no differences between groups with regard to AMH levels (0.63±0.59 *vs*. 0.94±0.97) and days of stimulation (9.57±1.87 *vs*. 10.29±2.82). Likewise, the levels of estradiol (788.94±670.82 *vs*. 940.16±694.69) and progesterone (617.29±319.76 *vs*. 561.18±486.78), as well as the number of follicles ≥18 mm (1.84±0.95 *vs*. 2.07±1.09) were similar in both groups.

**Table 1:** Demographic and treatment characteristics. Data expressed as mean ± DP

	Double lumen needle (n=105)	Single lumen needle (n=103)	*p*-value
Age	39.07±3.88	38.11±3.43	NS
Weight	61.73±17.53	65.96±15.44	NS
AMH	0.63±0.59	0.94±0.97	NS
Total Gonadotropins	1678.28±798.52	2080.45±852.36	0.0008
Stimulus duration	9.57±1.87	10.29±2.82	NS
Estradiol day hCG	788.94±670.82	940.16±694.69	NS
Progesterone day hCG	617.29±319.76	561.18±486.78	NS
Aspirated oocytes	3.00±2.11	3.69±2.20	0.02
M2 aspirated oocytes	2.20±1.64	2.99±1.88	0.02
Oocyte / follicle ratio	0.93	0.98	NS
Ratio of MII oocytes/ follicles	0.68	0.79	NS
Capture failure rate	16% (17/105)	9.8% (10/103)	NS
Fol 15-17mm day hCG	1.40±0.72	1.74±0.91	NS
Fol ≥18mm day hCG	1.84±0.95	2.07±1.09	NS

Despite the significant differences seen in total of gonadotropins (1678.28±798.52 *vs*. 2080.45±852.36; *p*=0.0008), number of aspirated oocytes (3.00±2.11 *vs*. 3.69±2.20; *p*=0.02) and metaphase II (2.20±1.64 *vs*. 2.99±1.88; *p*=0.02), indicators oocyte / follicle ratio ≥15 mm (0.93 *vs*. 0.98) and metaphase II oocytes / follicles ≥15 mm (0.68 *vs*. 0.79), closely associated with the main purpose of this study, were similar in both study groups. From a total of 208 punctures, oocytes were retrieved in 181 procedures, with a failure rate of 16.0% *vs*. 9.8%.

## DISCUSSION

In theory, routine follicular flushing provides for the retrieval of a greater number of oocytes, although it increases the duration of the procedure and extends exposure to anesthetics, to name a few potential harmful effects. We tried to evaluate the effectiveness of this practice in the retrieval of oocytes in patients characterized as poor responders.

Despite the apparent advantage of follicular flushing over direct aspiration, corroborated by relatively recent studies (Xiao *et al*., 2018; Souza *et al*., 2017), other authors have failed to consistently demonstrate the effectiveness of the procedure, whether in relation to the total number of oocytes, metaphase II oocytes, pregnancy or birth rates (Bagtharia & Haloob, 2005; Levy *et al*., 2012). Nevertheless, several centers still perform the procedure on a routine basis (Knight *et al*., 2001). Considering the arguments above, flushing would have no clinical value since its effectiveness in increasing the number of oocytes retrieved has not been proven.

Unlike other methodological models, our study did not assess cumulative retrieval rates, as Xiao *et al*. (2018) did, in which each aspiration of the same follicle was evaluated up to nine times. Although positive results were reported for follicular flushing, their retrospective study found a clear unfavorable impact, the duration of the procedure. In our series, patients submitted to flushing had a predetermined number of three aspirations, with data collected and recorded at the end of the procedure. Binary logistic regression analysis was not performed, since the possible effect of the number of flushes per procedure had not been studied.

Kumaran *et al*. (2015) specifically evaluated the pressure of the suction pump as a possible positive point in increasing the performance of the technique, with little data in medical literature on the subject. According to this group, the use of higher levels of pressure - 140 mmHg - was sufficient to produce better results. In contrast, our punctures were performed at a pressure of 90 mmHg, which might explain the difference in results and require additional inquiry. Still in this regard, a randomized controlled trial by Mok-Lin *et al*. (2013), although reporting similar levels of retrieved oocytes, described worse results in implantation and clinical pregnancy rates, attributing this curious and unexpected result to possible direct damage to oocytes from aspirated follicles, due to variations in intra-follicular pressure during the injection of the flushing medium.

Similarly to our results, a prospective randomized single-center trial by von Horn *et al*. (2017) evaluated 80 poor responders with fewer than 5 follicles >10 mm, and found that the aspiration group with three flushings did not show differences in the number of metaphase II oocytes, although duration was twice as long, a data not evaluated in our series. In the same direction, Haydardedeoglu *et al*. (2011) assessed 274 normal responsive patients, comprising 2,165 direct aspirations and 2,443 follicular flushing procedures, only to find similar results with respect to retrieved oocytes, metaphase II oocytes, oocytes in the germinal vesicle (GV) stage, although with significantly increased total aspiration time (8.26 *vs*. 12.52 minutes, respectively, *p* 0.01). The rates of fertilization, implantation, and good quality embryos were similar between the groups, differently from the study by Mok-Lin *et al*. (2013).

Our study was unable to demonstrate beneficial effects for aspiration with double-lumen needles in follicular flushing, as also reported in the most consistent randomized controlled studies available to date. Given the results presented by these RCTs and the absence of elements to indicate clear superiority of this procedure in normal and poor responders groups, it is our recommendation that direct aspiration with a single lumen needle be used as the standard procedure for all patients.
